# Experimental sexual selection reveals rapid evolutionary divergence in sex‐specific transcriptomes and their interactions following mating

**DOI:** 10.1111/mec.16473

**Published:** 2022-04-28

**Authors:** Paris Veltsos, Damiano Porcelli, Yongxiang Fang, Andrew R. Cossins, Michael G. Ritchie, Rhonda R. Snook

**Affiliations:** ^1^ Department of Ecology and Evolutionary Biology University of Kansas Lawrence Kansas USA; ^2^ Department of Animal and Plant Sciences University of Sheffield Sheffield UK; ^3^ 4591 CGR Institute of Infection, Veterinary and Ecological Sciences University of Liverpool Liverpool UK; ^4^ 4591 Centre for Genomic Research Institute for Integrative Biology University of Liverpool Liverpool UK; ^5^ 7486 Centre for Biological Diversity University of St Andrews St Andrews, Fife UK; ^6^ 7675 Department of Zoology Stockholm University Stockholm Sweden

**Keywords:** ejaculate‐female interactions, experimental evolution, female post mating response, reproduction, reproductive isolation, sexual conflict, Sfps

## Abstract

Post copulatory interactions between the sexes in internally fertilizing species elicits both sexual conflict and sexual selection. Macroevolutionary and comparative studies have linked these processes to rapid transcriptomic evolution in sex‐specific tissues and substantial transcriptomic post mating responses in females, patterns of which are altered when mating between reproductively isolated species. Here, we tested multiple predictions arising from sexual selection and conflict theory about the evolution of sex‐specific and tissue‐specific gene expression and the post mating response at the microevolutionary level. Following over 150 generations of experimental evolution under either reduced (enforced monogamy) or elevated (polyandry) sexual selection in *Drosophila pseudoobscura*, we found a substantial effect of sexual selection treatment on transcriptomic divergence in virgin male and female reproductive tissues (testes, male accessory glands, the female reproductive tract and ovaries). Sexual selection treatment also had a dominant effect on the post mating response, particularly in the female reproductive tract – the main arena for sexual conflict – compared to ovaries. This effect was asymmetric with monandry females typically showing more post mating responses than polyandry females, with enriched gene functions varying across treatments. The evolutionary history of the male partner had a larger effect on the post mating response of monandry females, but females from both sexual selection treatments showed unique patterns of gene expression and gene function when mating with males from the alternate treatment. Our microevolutionary results mostly confirm comparative macroevolutionary predictions on the role of sexual selection on transcriptomic divergence and altered gene regulation arising from divergent coevolutionary trajectories between sexual selection treatments.

## 1 INTRODUCTION

Sexual reproduction involves both pre‐ and post mating interactions between the sexes, and sexual selection influences male and female traits that mediate the fitness outcome of these interactions. While aspects of reproduction can be cooperative, the sexes can diverge over the optima of reproductive traits, such as courtship signals, fertilization and offspring production (Arnqvist & Rowe, 2005). The intensity of sexual selection is linked to the extent to which reproductive fitness optima differ between the sexes and can generate sexual antagonism, in which selection acts in opposing directions on the sexes (Holland & Rice, 1999; Rice, 1996). Comparative genomic studies have found that genes showing rapid divergence and stronger signatures of positive divergent selection are often sex‐biased or sex‐limited in expression (e.g., Cheng & Kirkpatrick, 2016; Ellegren & Parsch, 2007; Pröschel et al., 2006; Zhang et al., 2007). This is especially true of species showing signs of strong sexual selection, such as increased sexual dimorphism (Harrison et al., 2015; Wright et al., 2019).

In internally fertilizing species, the main arena for post ejaculatory molecular interactions is the female reproductive tract (FRT), which includes sites of sperm transfer, storage and subsequent fertilization of eggs transiting from the ovaries. Post mating female responses are extensive, influencing female behaviour, morphology and physiology. These responses are mediated by interactions between components of the male ejaculate, including sperm produced in the testes and seminal fluid proteins (Sfps) produced by accessory glands, and female reproductive proteins in the FRT and ovaries (Oku et al., 2019; Sirot et al., 2015; Wolfner, 2009). Post mating changes in females include altering investment in oogenesis (Wolfner, 2009), remating propensity (Chapman et al., 2003), and sperm storage and usage (Avila et al., 2010, 2015). Other aspects of female physiology are also altered, for example hunger (Carvalho et al., 2006), aggression (Bath et al., 2017) and physiological homeostasis (Cognigni et al., 2011; Ribeiro & Dickson, 2010). Genes associated with immunity and stress change gene expression upon mating in females, which may impact susceptibility or resistance to pathogens and/or parasites (Oku et al., 2019; Zhong et al., 2013).

An increasing number of studies have characterized transcriptomic post mating changes in females and the associated gene functions. Many studies typically examine either whole bodies or abdomens of females (Delbare et al., 2017; Fowler et al., 2019; Hollis et al., 2014, 2016; Innocenti et al., 2014; Innocenti & Morrow, 2009; Lawniczak & Begun, 2004; McGraw et al., 2008; Veltsos et al., 2017). Alternatively, some studies examine only one component of the FRT, either the “lower” reproductive tract, defined by the female sperm storage organs (Mack et al., 2006; Prokupek et al., 2008) or the “upper” reproductive tract, defined by the oviducts (Kapelnikov et al., 2008); but see (McDonough‐Goldstein et al., 2021). Receipt of the male ejaculate affects sperm storage dynamics, oogenesis and oviposition (Sirot et al., 2015) making them all subject to sexual selection (and sexual conflict). Likewise, for males, the main focus on the role of sexual selection and sexual conflict has been on Sfp evolution given that they are among the most rapidly evolving proteins known (Ahmed‐Braimah et al., 2017; Ellegren & Parsch, 2007). However, sperm and the cellular architecture of the testes also can be subject to rapid morphological evolution and sexual selection (Lüpold et al., 2009).

The strong post mating sexual selection and sexual conflict associated with the reproductive interactions between the sexes also cause rapid evolutionary changes between lineages, which could influence reproductive isolation (Ahmed‐Braimah et al., 2020; Manier et al., 2013; Markow, 1997). In particular, post mating prezygotic (PMPZ) reproductive isolation in which gametes do not interact properly prior to fertilization (Ahmed‐Braimah et al., 2017; Garlovsky et al., 2020) or affect egg production (Matute & Coyne, 2010) is hypothesized to result from divergent coevolutionary trajectories of sexual selection and sexual conflict in isolated populations. If different populations experience different population coevolution over time, then there will be gene expression or proteomic mismatches in sexual interactions between independently evolved lineages when the male ejaculate interacts with noncoevolved female reproductive tissues (Ahmed‐Braimah et al., 2020; Diaz et al., 2021; McCullough et al., 2020). However, such studies have focused on comparisons of the mating response of either heterospecific crosses or conspecific crosses where the sexual selection history of the populations are unknown.

The role of sexual selection in altering the coevolutionary dynamics between the sexes can be addressed experimentally. Experimental sexual selection manipulates the opportunity and strength of sexual selection by subjecting isolated populations to either polyandrous conditions, which promotes strong sexual selection, or enforced monandrous conditions, which reduces it. This approach has been used to examine the evolution of gene expression in response to sexual selection, and link it to macroevolutionary patterns of sex‐biased and sex‐limited gene expression evolution (Hollis et al., 2014; Immonen et al., 2014; Veltsos et al., 2017). These previous studies supported the role of sexual selection in divergent sex‐biased gene expression, but whether male‐ or female‐biased genes responded the most varies between species, sexes, tissues and sexual experience, for unknown reasons (Hollis et al., 2014, 2019; Immonen et al., 2014; Parker et al., 2019; Veltsos et al., 2017). Additionally, these studies were limited in that post mating responses and/or sex‐specific tissue responses were rarely examined. Consequently, understanding how sexual selection impacts sex‐limited and reproductive tissue‐specific gene expression, the consequences of this divergence on post mating responses, and whether such divergence results in altered regulation of gene expression when mating between sexual selection treatments is limited.

In this study, we used replicate populations of *D*. *pseudoobscura* after 150 generations of experimental evolution in which either monandry is enforced (referred to as M), which reduces sexual selection and conflict, or the opportunity of polyandry is elevated (referred to as E), which may increase the strength of selection and conflict, to test the hypotheses of the role of sexual selection on tissue‐specific and sex‐specific gene expression. Our previous study found substantial phenotypic responses to the manipulation of the sexual selection environment (Crudgington et al., 2005, 2009, 2010; Debelle et al., 2014, 2016; Snook et al., 2005), including those that could influence post mating responses such as investment in male accessory glands (Crudgington et al., 2009) and ovariole number and subsequent offspring production in females (Crudgington et al., 2010; Immonen et al., 2014). Sex‐biased gene expression evolution has also occurred but our previous studies were based on either whole bodies of only one sex (Immonen et al., 2014) or heads and abdomens of each sex in the virgin and courted condition, but not following mating (Veltsos et al., 2017). These phenotypic responses may result from evolution of tissue‐ and sex‐specific gene expression.

Here, a quantitative transcriptomic approach was made to investigate the impact of sexual selection on gene expression divergence, sampling male testes and accessory glands separately, and separating the FRT into the lower reproductive tract (including the uterus and sperm storage organs) and the ovaries. We first determined whether sexual selection treatment impacts virgin gene expression in all four tissues, testing the prediction that polyandry selects for upregulation. It has previously been suggested that males subjected to intense post copulatory sexual conflict should upregulate seminal fluid proteins for manipulation of female reproductive investment (Hollis et al., 2016). Likewise, polyandrous females should be poised for mating in anticipation of receipt of a manipulative male ejaculate that interacts within the FRT and thus should exhibit anticipatory upregulation of reproductive genes (Heifetz & Wolfner, 2004; Hollis et al., 2016; McGraw et al., 2004). We then determined the relative impact of sexual selection, mating per se, and their interaction for each female reproductive tissue. We tested the prediction that post mating interactions will diverge between sexual selection treatments, with poised polyandry females showing less upregulation upon mating relative to monandry females (see previous predictions). Such priming could be reflected in the types of genes that alter expression, including genes acting later during oogenesis (Immonen et al., 2014; Veltsos et al., 2017) and immune and stress response genes, all of which may show reduced changes in expression following mating in females already poised for mating. Immune and stress response genes have been commonly identified in female post mating transcriptomic responses, are assumed to indicate costs of mating, receipt of a foreign ejaculate and sexual conflict (Innocenti & Morrow, 2009; Zhong et al., 2013). Mating involves interactions between the sexes so we asked whether the post mating expression response of females arises from an interaction between the sexes or is primarily driven by one sex. Given that sexual selection is stronger on males (Winkler et al., 2021), we tested the prediction that polyandrous males will induce a larger female post mating transcriptomic response, especially with monandry females. Related, we tested the prediction that divergence of coevolutionary trajectories between monandry and polyandry populations will generate unique or more pronounced responses in crosses between these populations, which could form the basis of post mating prezygotic reproductive isolation.

## 2 MATERIALS AND METHODS

### 2.1 Experimental evolution lines

The origin, establishment, and maintenance of the selection lines are described in detail elsewhere (Crudgington et al., 2005). Briefly, 50 wild‐caught females of *D*.* pseudoobscura* from a population in Tucson, Arizona, USA were brought into the laboratory and reared for three generations, then four replicate lines of two different sexual selection treatments were established. We modified the opportunity for sexual selection by manipulating the adult sex ratio in food vials (2.5 × 80 mm) by either confining one female with a single male (enforced monogamy treatment; M, monadry) or one female with six males (elevated polyandry treatment; E, polyandry). This species is naturally polyandrous with wild‐caught females frequently being inseminated by at least two males at any given time (Anderson, 1974). We successfully equalized effective population sizes between the treatments (Snook et al., 2009). At each generation, offspring were collected and pooled together within each replicate line for each treatment, and a sample from this pool was used to start the next nonoverlapping generation in the appropriate sex ratios. Thus, this proportionally reflected the differential offspring production across families within a replicate and treatment. Generation time was 28 days and all populations were kept at 22°C on a 12L;12D cycle, with standard food media and added live yeast. Note that “monandry” versus “polyandry” as used here refers to the evolutionary history under which the individuals have evolved, not their current reproductive status.

### 2.2 Sample preparation

To generate experimental males and females, parents were collected from each replicate at generation 157–158. We standardized for maternal and larval environments as previously described (Crudgington et al., 2010). Briefly, parents were mated en masse in food bottles, transferred to containers with oviposition plates, allowed to oviposit for 24 h, and then 48 h later, 100 first instar larvae were seeded in standard food vials (Crudgington et al., 2010). Virgin males and females were collected under light CO_2_ anaesthesia on the day of eclosion and kept separate in vials of 10 individuals for 5 days to ensure reproductive maturity (Snook, 2001). On Day 5, within a 2 h window after lights turned on, one virgin female was placed in a food vial with one virgin male that was from either the same experimental replicate (“coevolved”; MM, EE where the first letter is the female) or the other treatment (“noncoevolved”; ME, EM). Our previous studies analysed gene expression in either whole body females or heads and abdomens of males and females. A potential criticism of this approach is that observed responses potentially confound changes in gene expression with allometric changes in relevant tissues (Montgomery & Mank, 2016). Most importantly, we know that male and female reproductive tissues are key to evolutionary responses to sexual selection and involved in sexual interactions. Therefore, here we carried out analyses of dissected male testes, accessory glands, and female ovaries and reproductive tracts (see Supporting Information S1 which illustrates the experimental design).

We dissected age‐ and circadian rhythm‐matched virgin males and females from the same collections. Each treatment was represented by 100 individuals, the tissues of which were equally split into 4 separate tubes, for easy pooling. Each pool contained the dissected tissues of the four biological replicates of the E or M treatments. For the mating treatments, males were put first in individual vials with fly food and allowed to settle. Females were then added, and were dissected 6 h after the first couple mated, in the order of mating, within a 2 h block. Dissections were performed under ether anaesthesia in RNAlater (Ambion) on ice blocks. We separately collected the ovaries and the remainder of the FRT, including the sperm storage organs (seminal receptacle and spermathecae). We refer to these different female tissue sets as ovaries and the FRT. The male accessory glands and testes were also dissected separately (ejaculatory bulbs were not included). All tissues were left at 4°C in RNAlater (Ambion) for one day and then transferred to –80°C until RNA extraction. The pools were processed for RNA extraction using Trizol (Ambion) following the manufacturer's instructions. RNA extractions were cleaned up in Qiagen RNeasy kit columns according to the manufacturer's protocol, including the 15 min DNase treatment. The quality of RNA extractions was checked with Nanodrop and Bioanalyser.

### 2.3 Sequencing and mapping

Illumina libraries were prepared using the ScriptSeq kit (Illumina Inc) following the manufacturer's protocol. rRNA was depleted using Epicenters’ Ribozero kit. Paired‐end second stranded libraries were sequenced at 100 base pair (bp) read length using an Illumina HiSeq 2000. Reads were mapped to the *D*. *pseudoobscura* genome v3.1, and indexed using bowtie2 (Langmead et al., 2009). Paired‐end reads were aligned using option “‐g 1 –library‐type fr‐secondstrand” with TopHat2.0.8b (which calls bowtie2.1.0; Kim et al., 2013) and instructs TopHat2 to report the best alignment to the reference. Exon features were counted using HTSeq‐count (Anders et al., 2015) and the reads of all exons of each gene were combined to provide overall measures of gene expression (Veltsos, 2021).

### 2.4 Statistical analysis

We analysed the count data using edgeR v3.18.1 (Robinson et al., 2010) running in R v.3.4.0 (R Development Core Team, 2007) with scripts from (Veltsos, 2021). The tissues were always analysed separately but, for statistical analysis of specific contrasts, all libraries for each tissue were used, including libraries not contributing to that contrast. This was to allow incorporating as much information as possible on gene expression variance, counteracting the fact that data points available for each gene in a specific contrast are limited. Libraries were normalised with the TMM procedure in edgeR. Because the analysis was performed for each tissue separately, not all annotated genes had counts in each analysis. Additionally, normalisation can result in negative counts for some genes. Therefore, for analysis we considered only genes with average 0 normalized counts per million across all libraries used in each analysis were retained. The number of genes retained for analysis were therefore 11,751 genes for testes, 11,754 for accessory glands, 10,272 for female reproductive tracts and 8624 genes for ovaries out of 16,467 annotated genes for *D*. *pseudoobscura*. Dispersion was measured with default parameters using a negative binomial model. We considered genes to be differentially expressed (DE) if they were below the 5% false discovery rate (FDR) threshold (Benjamini & Hochberg, 1995). We did not employ a log_2_FC threshold because allometry is unlikely to influence results obtained from specific tissues (Montgomery & Mank, 2016).

We made specific statistical contrasts in the gene expression profiles in order to address the evolutionary hypotheses raised in the introduction. First, we assessed whether the strength of sexual selection impacts the evolution of gene expression in virgin male reproductive tissues, by detecting differential expression in the contrast E versus M (Supporting Information S2a,b). Second, we addressed the same question in a similar manner for female reproductive tissues (Supporting Information S2c,d). Third, we investigated the effect of sexual selection history on the female mating response by analysing the contrasts equivalent to the main effect of sexual selection history (E + EE vs. M + MM, where single letters indicate virgin status and double letters indicate mated status with the female partner written first), the main effect of mating (E + M vs. EE + MM), and their interaction (E + MM vs. M + EE). We report these results by considering the virgin gene expression status to be the baseline when compared to mated females. To separate the effects of the male and female sexual selection history on the female mating response we ran a fourth model in which we contrasted the main effect of female treatment (EE + EM vs. MM + ME), the main effect of male treatment (EE + ME vs. MM + EM) and their interaction (EE + MM vs. EM + ME). For this analysis we consider the coevolved mating as a baseline and categorize the noncoevolved post mating response as having either relatively higher (EM or ME up) or lower (EM down or ME down) expression.

**FIGURE 1:**
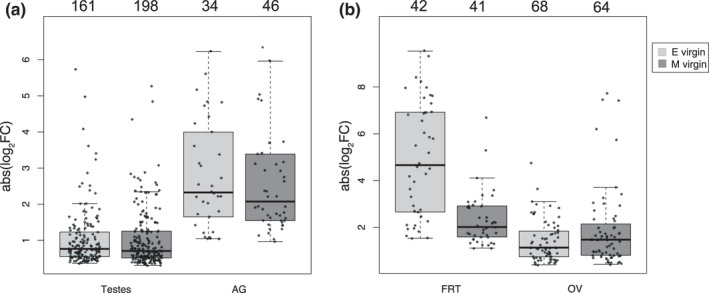
Differential gene expression (absolute log_2_FC changes, *y*‐axis) in virgin (a) male or (b) female tissues comparing responses of upregulated genes in either polyandry (E: light grey) or monandry (M: dark grey) selection treatments. Dots indicate differentially expressed (DE) genes and their number is noted above each box

**FIGURE 2:**
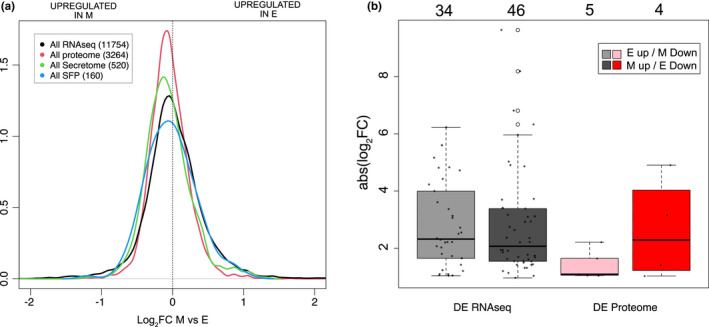
Effect of sexual selection on expression of genes coding for (a) all and (b) differentially expressed male accessory gland genes between polyandry (positive *x*‐axis values) and monandry (negative *x*‐axis values) treatments. Line colours in a) differentiate between all accessory gland genes (black) or those categorized as proteome (red), secretome (green) or seminal fluid proteins (blue) based on Karr et al. (2019). The number of genes is indicated in the (a) parentheses and (b) above the boxes. Bar colours (b) differentiate the number (dots) and magnitude of absolute gene expression changes (*y*‐axis) for all differentially expressed (DE) male accessory gland genes (All RNAseq; grey) or for the DE accessory gland proteome genes (Proteome; coloured) showing either elevated expression in E (light grey or pink) or M (dark grey or red) males. NB: secretome and SFP genes were not DE [Colour figure can be viewed at wileyonlinelibrary.com]

We tested the significance of proportions of significantly up‐ and downregulated genes in each contrast for departure from a 50% expectation using the chisq.test function in R. When comparing the number of differentially expressed (DE) genes across virgin contrasts (Figures 1 and 2) which had different total numbers of genes in each contrast, we performed chisq.tests on their proportion across all genes expressed in both contrasts. For example, in Figure 1a we tested 359/11,751 genes expressed in testes versus 80/11,754 genes expressed in accessory glands. Finally, when comparing numbers of DE genes in the coevolved and noncoevolved treatment subsets separately for E and M mated females (Figure 4), we tested against a 50% expectation since the total number of possible genes is the same in all contrasts. This is because the contrasts are part of a model that included all six types of libraries for E and M females of virgin, coevolved and noncoevolved mating status. Differences in gene expression magnitude of genes within a contrast were texted using the wilcox.test function in R.

We performed gene ontology (GO) enrichment analysis using topGO v2.22.0 with the weight01 algorithm to account for GO topology (Alexa & Rahnenfuhrer, 2010). The GO universe was defined from the genes that showed appreciable expression in each tissue. Results with *p* < .05 on Fisher's exact tests, corrected for topology, were retained (Supporting Information S3).

For analysis of Sfps, we contrasted the distribution of the change in expression (log_2_FC) of all genes, and DE genes in the contrast between sexual selection treatments for virgin accessory gland transcriptomes using density plots and boxplots, respectively. We compared the distributions of three Sfp‐related gene subsets, identified from *D*. *pseudoobscura* proteomics (Karr et al., 2019). The largest subset was 3,281 proteins produced in the accessory gland (“proteome”). Of these, 528 had protein secretory signals (“secretome”) and 163 were also orthologous to *D*. *melanogaster* seminal fluid proteins (putative Sfps or “exoproteome”). The majority of these genes could be cross‐referenced to our data (Figure 2 legend), but only proteome genes were detected among the DE accessory gland genes.

## 3 RESULTS

### 3.1 Sexual selection causes gene expression divergence in virgin male reproductive tissues

Previous work has hypothesized that monandry selects for downregulation and polyandry selects for upregulation of genes expressed in male reproductive tissues in the abdomen of *D*. *melanogaster* (Hollis et al., 2016). We found that sexual selection history caused divergence in gene expression in virgin male testes for 359 DE genes. However, contrary to prediction, there were marginally more upregulated genes in monandry (Figure 1a; *χ* ^2^ = 3.81, degrees of freedom [*df*] = 1, *p* = .051; Supporting Information S2). Those 198 genes were related to the biological processes (BP) of proteolysis, digestion and the innate immune response (Supporting Information S3; tab “testis EvsM”). Τhe 161 upregulated genes in polyandry males had BP enrichment related to stress responses, such as double strand break repair, cellular response to UV, and terpenoid metabolism (Supporting Information S3; tab “testis EvsM”).

In male accessory glands, 80 genes were DE between sexual selection treatments but there was no difference in the number of upregulated genes between treatments (Figure 1a; *χ* ^2^ = 1.8, *df* = 1, *p* = .18). The 34 accessory gland genes upregulated in polyandry males were enriched for BP terms related to eggshell chorion assembly, development, and neuropeptide signalling (Supporting Information S3; tab “agland EvsM”) whereas the 46 genes upregulated in monandry males were enriched for the BP term “detection of chemical stimulus involved in sensory perception” (Supporting Information S3; tab “agland EvsM”). None of the DE genes in accessory glands were putative Sfps (Karr et al., 2019).

The rapid evolution of Sfps, both genomically and transcriptomically, is usually thought to reflect strong sexual selection. However, we found a larger proportion of testes genes were differentially expressed compared to accessory gland genes (359/11,751 vs. 80/11,754; *χ* ^2^ = 179.47, *df* = 1, *p* < .001). The magnitude of gene expression within each tissue did not differ between monandry and polyandry males, as might be expected under the “poised” hypothesis, for example (testis: *W* = 14,712, *p* = .21; accessory glands: *W* = 752, *p* = .78; Figure 1a). To examine whether Sfps had higher expression in polyandrous males, as predicted (Hollis et al., 2016), we compared expression levels between the sexual selection treatments of different sets (proteome, Sfps, and secretome genes) of accessory gland proteins recently described for *D*. *pseudoobscura* (Karr et al., 2019). Results did not support the prediction; monandry males had higher overall expression of secretome proteins compared to polyandry males (*W* = 21,345,000, *p* < .001; Figure 2a) and sexual selection treatment did not affect gene expression of proteome or Sfp genes. Examination of the 80 accessory gland genes that were differentially expressed between treatments, regardless of set, showed we found only a nonsignificant trend towards higher expression in polyandry males (Figure 2b).

In conclusion, virgin male reproductive tissue expression was affected by experimental manipulation of the strength of sexual selection but not in the predicted direction. Both the total number and proportion of DE genes was higher in testes than accessory glands. There was also a weak trend for monandry, not polyandry, males to upregulate testes genes and accessory gland genes with secretory signals.

### 3.2 Sexual selection causes gene expression divergence in virgin female reproductive tissues

Similar to males, we tested whether monandry selects for downregulation and polyandry selects for upregulation of genes (Hollis et al., 2016) in virgin female reproductive tissues (FRT and ovaries), such that polyandry females are more poised for subsequent mating. While there was an effect of sexual selection on gene expression in both tissues, the number of genes upregulated in each sexual selection treatment did not differ (Figure 1b; FRT: *χ* ^2^ = 0.01, df = 1, *p* = .9; ovaries: *χ* ^2^ = 0.12, *df* = 1, *p* = .73). In the FRT, 42 genes were upregulated in polyandry and were enriched in several BP terms associated with the immune system whereas 41 genes upregulated in monandry were enriched for insemination (Supporting Information S3; tab “FRT EvsM”). In ovaries, the 68 genes upregulated in polyandry, but not the 64 genes upregulated in monandry, were enriched in BP terms associated with eggshell chorion assembly (Supporting Information S3, tab “ovEvsM”).

The proportion of DE genes in ovaries was significantly greater than in the FRT (132/8625 vs. 83/10,273; *χ* ^2^ = 21.12, *df* = 1, *p* < .001). Even though there was no difference in the number of upregulated genes between sexual selection treatments, the magnitude of gene expression was impacted in a tissue‐specific way. In the FRT, but not ovaries, logFC of upregulated genes in polyandry was greater than in monandry (FRT: *W* = 302, *p* < .001; ovaries: *W* = 2452, *p* = .21; Figure 1b).

Overall, we found that experimental sexual selection altered gene expression and, while there was no treatment bias in number of responding DE genes, FRTs had a higher magnitude of upregulation in polyandry compared to monandry. Moreover, the reproductive tissues responded differently, with a higher percentage of ovarian genes changing in expression compared to the FRT. These results support the hypothesis that polyandry females are more poised for mating via increases in expression magnitude in the FRT, the primary site of molecular interactions between the female and male ejaculate.

### 3.3 Sexual selection causes divergence in the female post mating response

With regard to the female post mating response, we expected polyandry females to already express post mating response genes as virgins, whereas monandry females would increase the expression of these genes after mating (the poised hypothesis; Heifetz & Wolfner, 2004; McGraw et al., 2004). This response would be seen as a significant selection treatment by mating status interaction in our model (Table 1).

**TABLE 1:** Outcome of contrasts testing the effect of sexual selection, mating status, and their interaction on the female post mating response in the female reproductive tract (FRT) and ovaries

	Effect	Contrast	Total gene number	Higher expression for first contrast term	Higher expression for second contrast term	*χ* ^2^	*p*‐value
Female reproductive tract (FRT)	Sexual selection	E + EE vs. M + MM	129	35	94	27.0	**<.001**
	Mating	E + M vs. EE + MM	244	87	157	20.1	**<.001**
	Sexual selection × mating	E + MM vs. M + EE	146	143	3	134.3	**<.001**
Ovaries	Sexual selection	E + EE vs. M + MM	604	270	334	6.78	**<.01**
	Mating	E + M vs. EE MM	43	17	26	1.88	.17
	Sexual selection × mating	E + MM vs. M + EE	0	0	0		

Note

Degrees of freedom are always 1. Chi‐square tests were performed against the null hypothesis of 50% for the number of upregulated genes between the first and second contrast terms. Statistically significant results are indicated in bold. NA indicates the numbers were too low to meaningfully statistically compare. Single letter (E, M) contrast names indicate virgins, two letters (EE, EM, ME, MM) indicate mated with the female partner written first.

**FIGURE 3:**
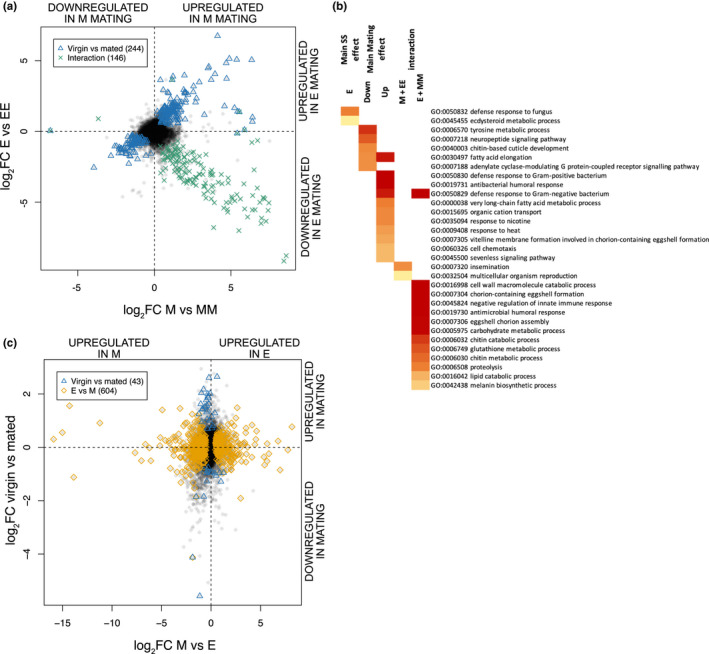
Post mating gene expression (log_2_FC) responses and enriched gene ontology (GO) biological process (BP) terms in female reproductive tissues for (a) differentially expressed (DE) genes in female reproductive tracts (FRT), (b) BP term enrichment for significant factors, and (c) DE genes in ovaries. DE genes are indicated with blue triangles for the main effect of mating status, yellow diamonds for the main effect of sexual selection treatment and green crosses for the interaction between the two. In (a) the axes show the coevolved post mating response for each sexual selection treatment, which allows visualisation of the interaction between sexual selection treatment and mating as the diagonal on which the green crosses fall. In (c) the axes represent the two main effects (note change in scale), as there were no significant interaction effects. The number of DE genes is indicated in parentheses. See Table 1 for statistics [Colour figure can be viewed at wileyonlinelibrary.com]

For the FRT, we found both the main effects of sexual selection treatment and mating, and their interaction, to be significant (Table 1). To illustrate the interaction, the main effect of mating within each female treatment was plotted on separate axes, and the genes that always respond to mating and those that show significant interaction effects between mating and sexual selection treatment are indicated separately (Figure 3a). Most (143/146) of the significant genes for the interaction were upregulated upon mating in monandry females (and downregulated upon mating in polyandry females; Table 1; Figure 3a [bottom right quadrant]). These genes were enriched for immune function, a variety of metabolic processes, and eggshell chorion assembly (Figure 3b; Supporting Information S3; tab “SS treatment x mating FRT”). The three genes showing upregulation in mated polyandry and downregulation in monandry have BP terms related to reproduction (Figure 3b; Supporting Information S3; tab “SS treatment x mating FRT”). Of the 244 DE genes responding to mating regardless of sexual selection history, more were upregulated after mating than downregulated (Table 1, Figure 3a). The commonly upregulated genes after mating were enriched for BP terms associated with stress and immune responses, only one of which was shared with BP enrichment of genes significant for the interaction (Figure 3b; Supporting Information S3; tab “Main effect of mating FRT”). Genes downregulated after mating were not enriched for BP related to immune/stress responses (Figure 3b; Supporting Information S3; tab “Main effect of mating FRT”). With regard to the main effect of sexual selection treatment (Table 1), 94 genes were upregulated in monandry (but showed no BP enrichment) and only 35 genes were upregulated in polyandry (with two enriched BP terms – defence response to fungus and ecdysteriod metabolic process; Supporting Information S3; tab “Main effect of SS treatment FRT”; Figure 3b).

In the ovaries, only the main effect of sexual selection treatment was associated with differential gene expression. As with the FRT, there were more upregulated genes in monadry versus polyandry (Table 1; Figure 3c). The 334 genes upregulated in monandry females were enriched in BP terms related to tissue development whereas the 270 genes upregulated in polyandry were enriched for eggshell chorion assembly (Supporting Information S3; tab “Main effect of SS treatment OV”).

Thus, analyses for both the FRT and the ovaries suggest that polyandry females are more poised for mating based on reduced number of DE genes after mating. This interpretation is further supported by BP enrichment of upregulated genes under polyandry that relate to egg production in ovaries and stress and immune responses in the FRT. The latter may attest to polyandry males being potentially immunogenic (Innocenti & Morrow, 2009).

### 3.4 Female, not male, sexual selection treatment drives the female post mating gene expression response

The post mating gene expression response in female reproductive tissues represents an interaction between the sexes. To examine these interactions in more detail, we partitioned gene expression in females mated to males of the same or different sexual selection treatment, determining the effect of female treatment, male treatment, and their interaction. The interaction tests whether matings between coevolved individuals with respect to sexual selection treatment respond differently than matings between noncoevolved individuals (Table 2). Since the strength of sexual selection is stronger in males than females (Winkler et al., 2021), we predicted that both the male effect and the interaction would have the strongest impact on the female post mating response (Table 2).

**TABLE 2:** Outcome of contrasts testing the effect of female sexual selection treatment, male sexual selection treatment, and their interaction on the female post mating response in the female reproductive tract (FRT) and ovaries

	Effect	Contrast	Total gene number	Higher expression for first contrast term	Higher expression for second contrast term	*χ* ^2^	*p*‐value
Female reproductive tract (FRT)	Female	EE + EM vs. MM + ME	198	38	160	75.2	**<.001**
	Male	EE + ME vs. MM + EM	7	0	7	NA	NA
	Female × Male	EE + MM vs. EM + ME	0	0	0	NA	NA
Ovaries	Female	EE + EM vs. MM + ME	566	306	260	3.74	.**053**
	Male	EE + ME vs. MM + EM	0	0	0	NA	NA
	Female × Male	EE + MM vs. EM + ME	6	4	2	NA	NA

Note

Degrees of freedom are always 1. Chi‐square tests were performed against the null hypothesis of 50% for the number of upregulated genes between the first and second contrast terms. Statistically significant results are indicated in bold. NA indicates the numbers were too low to meaningfully statistically compare. Single letter (E, M) contrast names indicate virgins, two letters (EE, EM, ME, MM) indicate mated with the female partner written first.

Contrary to predictions, in both female tissues, we found no significant interaction effect and little to no male effect (Table 2). There was a small male effect in the FRT with seven DE genes, enriched for egg chorion BP, all upregulated more in females mated with monandry males (Table 2; Supporting Information S3; tab “Main male effect FRT”). Surprisingly, for both the FRT and ovaries, the female effect was stronger and the sexual selection effect asymmetric between tissues. Of the 198 DE genes in FRT, 160 were upregulated more when monandry females mated because they were more highly expressed in mated monandry than polyandry females (Table 2) and are enriched for immune response and metabolic processes, while the 38 genes upregulated more in mated polyandry females did not show any significant enrichment (Supporting Information S3; tab “Main female effect FRT”). In contrast, in the ovaries, of the 566 DE genes, 306 were marginally more upregulated in mated polyandry females and these genes were enriched for eggshell chorion assembly BP. The 260 genes upregulated in mated monandry females did not have GO enrichment terms consistent for any clear function (Supporting Information S3; tab “Main female effect OV”).

In summary, female sexual selection history plays a critical role in determining the extent and function of differences in the post mating response, with the effect of male and interaction between the sexes minimal. Again, mated polyandry females had relatively suppressed gene expression changes relative to mated monandry females in the FRT. However, in the ovaries, mated polyandry females had relatively more highly expressed genes although these were related to egg production, perhaps suggesting that polyandry females can “gear up” for oogenesis more quickly than monandry females. One caveat to this analysis is that it combines females from the two sexual selection treatments to test for male and female effects. Given that we showed above that sexual selection treatment influences the female post mating response, consequences of interactions between males and females may be obscured.

### 3.5 Sexual selection asymmetrically alters post mating gene expression

To further decompose the effect of sexual selection treatment origin on the female post mating response, we examined gene expression in coevolved and noncoevolved matings separately for females of different sexual selection history. This also allowed us to test the prediction that divergent coevolutionary trajectories between sexual selection treatments would generate unique or more pronounced responses when mating with a noncoevolved male. We predict this effect will be asymmetric as monogamous females have not evolved in a highly manipulative environment in response to high male competition.

In the ovaries, both polyandry and monandry females had limited post mating changes in gene expression, regardless of the sexual selection treatment of the male partner (Table 3). Combined with the insignificant overall effect on mated females (Table 2), this suggests that the males have only a small, if any, effect on the post mating response of ovaries in monandry and polyandry females. In contrast, patterns were varied in the FRT, in which monandry females showed significant gene upregulation following mating, regardless of mating partner (Table 3) whereas in polyandry females there was no significant differential post mating response based on sexual selection treatment of the male (Table 3).

**TABLE 3:** Outcome of contrasts testing the effect of male origin on the female post mating response in the female reproductive tract (FRT) and ovaries for monandry and polyandry females

	Effect	Contrast	Total gene number	Higher expression for first contrast term	Higher expression for second contrast term	*χ* ^2^	*p*‐value
Female reproductive tract (FRT)	Monandry coevolved	M vs. MM	160	57	103	13.3	**<.001**
	Monandry noncoevolved	M vs. ME	363	157	206	6.61	**<.01**
	Polyandry coevolved	E vs. EE	199	99	100	0.005	.94
	Polyandry noncoevolved	E vs. EM	90	51	39	1.6	.21
Ovaries	Monandry coevolved	M vs. MM	1	0	1	NA	NA
	Monandry noncoevolved	M vs. ME	3	1	2	NA	NA
	Polyandry coevolved	E vs. EE	6	1	6	NA	NA
	Polyandry noncoevolved	E vs. EM	0	0	0	NA	NA

Note

Degrees of freedom are always 1. Chi‐square tests were performed against the null hypothesis of 50% for the number of upregulated genes between the first and second contrast terms. Statistically significant results are indicated in bold. NA indicates the numbers were too low to meaningfully statistically compare. Single letter (E, M) contrast names indicate virgins, two letters (EE, EM, ME, MM) indicate mated with the female partner written first.

**FIGURE 4:**
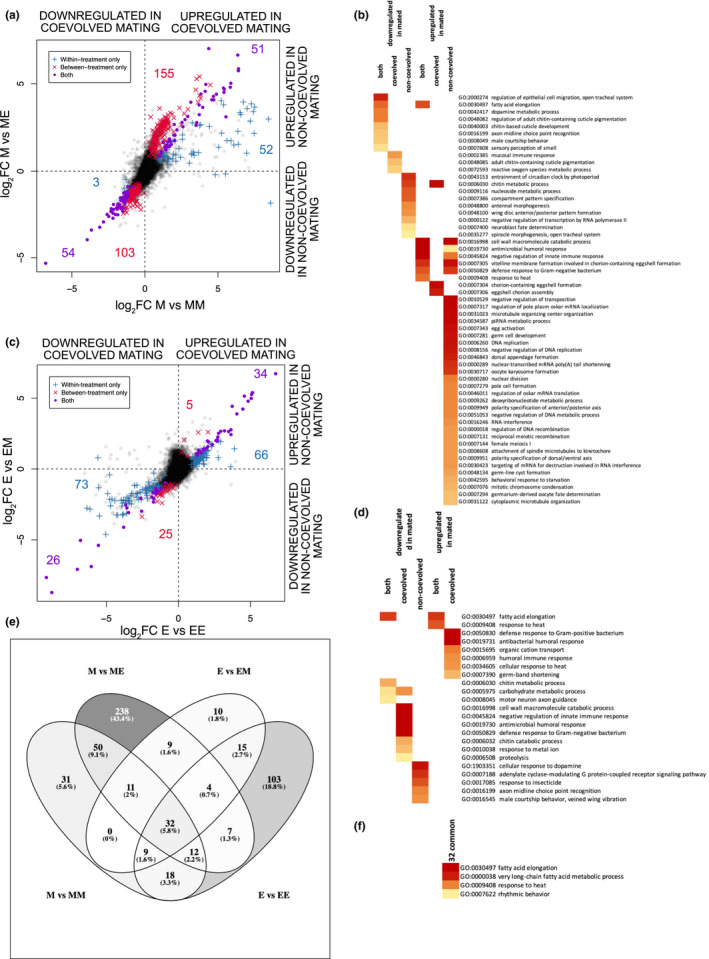
Post mating responses of differentially expressed (DE) genes (log_2_FC) in the female reproductive tracts (FRT) of (a) monogamy and (c) polyandry females when mated to males either from the same sexual selection treatment or the opposite treatment. Genes are categorized as being DE only in coevolved mating (blue +), only in noncoevolved mating (red x) or regardless of male treatment (purple o). In each plot, the *x*‐axis represents is the coevolved post mating response and the *y*‐axis the noncoevolved post mating response with negative values representing virgin‐biased genes and positive values representing mating‐biased. The coloured numbers within the plots are the number of DE genes for each gene category. (b) and (d) Represent the associated gene ontology (GO) biological process (BP) enrichment of the DE genes of panels (a) and (c), respectively. (e) Shows the overlap of genes corresponding to coevolved and noncoevolved mating separately in monogamy and polyandry females, and (f) shows the GO BP enrichment of the 32 consistently DE genes all (e) contrasts [Colour figure can be viewed at wileyonlinelibrary.com]

Divergent coevolutionary trajectories between sexual selection treatments may result in unique gene expression between coevolved and noncoevolved crosses, irrespective of the total number of genes that alter expression. Indeed, while we found some genes alter gene expression in the same direction regardless of male mate origin, many genes are uniquely expressed dependent on male origin and this is asymmetric across sexual selection treatments. For monandry females, more unique genes change expression when mating with polyandry males than when mating with coevolved males (coevolved = 55 vs. noncoevolved = 258; *χ* ^2^ = 131.66, *p* < .001; Figure 4a) but the opposite pattern occurs in polyandry females (coevolved = 139 vs. noncoevolved = 30; *χ* ^2^ = 70.30, *p* < .001; Figure 4c). Comparing the female sexual selection treatments, mating with noncoevolved males caused more genes to alter expression in monandry females than in polyandry females (monandry = 258 vs. polyandry = 30; *χ* ^2^ = 180.50, *p* < .001; Figure 4a,c). There was no difference in the proportion of genes that had equal responses in both M and E females regardless of partner (the purple points that fall on the diagonal in Figure 4a,c; 25.1% vs. 26.2%, *χ* ^2^ = 0.04, *p* > .05). Regardless of DE number, uniquely DE genes for noncoevolved crosses show a unique pattern of gene expression for both sexual selection treatments (Figure 4; compare the red x's to the purple dots and blue crosses), which generally do not overlap with either shared or uniquely coevolved responses.

Given novel genes were altered in expression when mating with noncoevolved males, we also examined GO terms for the genes that differ in expression between coevolved and noncoevolved crosses. For monandry females, many of the BP terms for uniquely upregulated genes after noncoevolved mating are related to DNA replication and germ cell development, and downregulated noncoevolved responses are related to morphogenetic processes (Figure 4b; Supporting Information S3; tab “venn FRT M”). Genes affected by the male partner in polyandry females had few BP enrichment terms with unclear biological interpretation (Figure 4b; Supporting Information S3; tab “venn FRT E”). Moreover, the identity of post mating response genes differs between monandry and polyandry females, including when mated with noncoevolved males (Figure 4e). Only 32 DE genes are shared across all four mating combinations, suggesting that these are critical to female post mating responses (Figure 4e; represented by some of the purple dots in Figure 4a,c). These shared genes are enriched for four BP processes associated with fatty acid production, stress response and rhythmic behavior (Figure 4f; (Supporting Information S3; tab “venn 32”).

Overall, these results confirm predictions, highlighting consistent asymmetric female post mating responses between sexual selection treatments. Results suggest monandry females are less resistant to male manipulation and/or polyandrous males are more manipulative and/or polyandrous females are more resistant, that differentially regulated genes in coevolved and noncoevolved matings showed little overlap in enriched BP terms between females of the different sexual selection treatments, and that, despite differences in total number and function, noncoevolved responses had different expression patterns than coevolved crosses.

## 4 DISCUSSION

Understanding how sexual selection impacts the molecular basis of sexual interactions is important because it can have profound effects on sex‐specific fitness and is predicted to influence the evolution of postmating prezygotic reproductive isolation. Here, we combined transcriptomics with experimental evolution to determine how sexual selection affects gene regulation simultaneously in multiple sex‐specific tissues in male and female virgins and in the post mating female response when mating with either males that evolved under the same or different sexual selection treatment. Sexual selection and sexual conflict should result in polyandry males that are manipulative and females that are resistant whereas enforced monogamy should reduce male manipulation and female resistance (Arnqvist & Rowe, 2005). We tested whether signatures of these predictions can be identified in changes in gene regulation over the time frame of 150 generations of experimental manipulation of sexual selection and whether short‐term changes in the female post mating response also diverges.

We tested the prediction that polyandry selects for upregulation in virgin male and female reproductive tissues (Heifetz & Wolfner, 2004; Hollis et al., 2016; McGraw et al., 2004). Given the role of accessory glands in manipulating female reproductive investment, and that the FRT is the main site of molecular interactions between the sexes, we expected that these tissues would show more divergent gene regulation than testes or ovaries. Support for these predictions varied. In males, sexual selection resulted in divergent gene regulation in both testes and accessory glands. Contrary to predictions, a larger proportion of genes change expression in the testes and relaxation of sexual selection resulted in more upregulated genes in the testes and higher expression of accessory gland genes with a signal of a secretory function. However, the function of genes that changed expression in the testes and accessory glands were distinct for each sexual selection treatment, suggesting differences in the potential consequences on male reproductive fitness and effects on female mates. In females, sexual selection resulted in divergent gene regulation in both the FRT and ovaries, but a larger proportion of ovary genes were differentially expressed compared to the FRT. These results are contrary to predictions. However, the magnitude of differential gene expression is larger in the FRT and for polyandry females, which supports the prediction. We previously showed that virgin polyandry *D*. *pseudoobscura* females have more ovarioles than monandry females (Immonen et al., 2014). Using female whole body microarrays, we found upregulated genes in polyandry females to be associated with oogenesis (and likely to be expressed in the ovary) whereas upregulated genes in monandry were associated with genes in somatic tissues and metabolism (Immonen et al., 2014). In the current analysis, virgin polyandry females had upregulated genes with functions in immune responses and later stages of egg production whereas virgin monandry females upregulated genes associated with BMP signalling pathway which is involved in early patterning the *Drosophila* eggshell (Niepielko et al., 2012). These results support the hypothesis that polyandry females are poised for receipt of a potentially manipulative ejaculate (Heifetz & Wolfner, 2004; McGraw et al., 2004).

We also examined the female post mating response testing the effect of sexual selection, mating and their interaction on gene expression divergence. In particular, we tested the prediction that polyandry females would show a smaller post mating response relative to monandry females given polyandry females are poised for mating. We additionally asked whether post mating responses are specific to sexual selection treatment with respect to biological processes. As predicted, we found an interaction between sexual selection treatment gene expression and mating status but this was tissue specific, occurring only in the FRT, and asymmetric across sexual selection treatments, with most DE genes upregulated in mated monandry females. Moreover, we saw a contrasting pattern in that genes which are upregulated upon mating in monandry females had lower expression in mated polyandry females, supporting the hypothesis that polyandry selects for females to be poised for mating, perhaps to combat a manipulative ejaculate. While gene expression in ovaries showed no interaction effect, monandry females also upregulated more genes after mating than polyandry females, with gene functions supporting more advanced reproductive development in polyandry females (also supported in Immonen et al., 2014; Veltsos et al., 2017). Overall, these results support the hypothesis that polyandry females are poised for mating. In the FRT, DE genes associated with the interaction effect and for mating status were enriched for immune function, a commonly observed effect of mating in Drosophila (Hollis et al., 2019; Innocenti & Morrow, 2009; Sirot et al., 2015). It has previously been suggested that upregulation of these genes arises from sexually antagonistic interactions between the sexes, such that males are immunogenic to females (Innocenti & Morrow, 2009; Zhong et al., 2013). However, there remains insufficient data to determine whether these effects are detrimental or beneficial to females overall (Bagchi et al., 2021; Oku et al., 2019).

Given that sexual selection changes the pattern of gene expression in mated females in both female reproductive tissues, that the female post mating response arises as an interaction between the sexes, and that sexual selection is stronger on males (Winkler et al., 2021), we expected that effects of male treatment and interactions should drive the female post mating transcriptomic response. This should be particularly prominent in the FRT, as the main arena for between‐sex reproductive molecular interactions. We tested these predictions by determining the relative contributions of females, males and interactions between the sexes on the pattern of gene expression in mated females. However, disentangling male and female effects (the latter of which will include differences between virgins) is complex within each sexual selection treatment so, to test these predictions, we crossed males and females both within and between sexual selection treatments. Contrary to predictions, we found no interactions and little male effect, in both the FRT and ovaries. The dominant or only effect was attributed to female sexual selection treatment and this effect varied between tissues. The FRT of monandry females showed significantly more upregulated genes (enriched for immune function) than for polyandry females whereas, in the ovaries, polyandry females upregulated significantly more genes (enriched for later stage egg production) than monandry females. These results are consistent with our other analyses showing that polyandry females are more poised for mating and reproduction with little effect of male partner.

This latter result is surprising and may be confounded by the test performed, which combines non‐coevolved and coevolved crosses in estimating each sex effect. We independently tested the monandry and polyandry female post mating response based on male partner origin to test the hypothesis that gene expression divergence, mediated by variation in sexual selection between isolated populations, generates potential reproductive mismatches via altered gene expression regulation of female post mating responses. Moreover, given that monandry should relax male manipulation and female resistance, we tested the prediction that altered regulation when mating with a noncoevolved male would be asymmetric across the different sexual selection treatments and, because of different evolutionary trajectories of monandry and polyandry populations, target different types of genes between the sexual selection treatments. Such responses may eventually generate post mating prezygotic (PMPZ) reproductive incompatibilities. Several studies have compared post mating transcriptomic responses to test the idea of reproductive mismatches generating PMPZ (Ahmed‐Braimah et al., 2020; Bono et al., 2011; McCullough et al., 2020). However, it is difficult to infer the historical role of different evolutionary processes from patterns of contemporary divergence between species and therefore whether mismatches generated PMPZ or evolved after divergence cannot be determined. Experimental evolution can address this problem as it can distinguish between current and historical processes, but lacks the full complexity of natural conditions and typically does not result in reproductive isolation over the relatively short time frame studied.

Using experimental sexual selection, we found patterns that support these predictions in the FRT, but not in ovaries. Gene regulation in the FRT varied between monandry and polyandry females and depended on the type of male involved. We inferred altered regulation by the identification of uniquely DE genes when mated with a noncoevolved male and comparing their prevalence with uniquely DE genes when mated with a coevolved male. Both monandry and polyandry females had more unique DE genes when mated to polyandry males than monandry males, suggesting polyandry males can manipulate female gene expression. Monandry females exhibit more DE genes when mating with polyandry males, suggesting that monandry females are less resistant to male manipulation, as predicted. The uniquely upregulated genes in monandry females from mating with a noncoevolved male were associated with DNA replication and germ cell development suggesting polyandry males manipulate investment in reproduction. Interestingly, the fewer genes affected by the male partner in polyandry females showed no clear affected process. Finally, uniquely DE genes from noncoevolved crosses showed different expression patterns than shared or uniquely DE coevolved patterns as would be expected given no recent coevolutionary history. It remains to be determined whether continued divergence and these mismatched gene regulations would generate PMPZ.

In conclusion, our results tested several predictions arising from sexual selection and sexual conflict theory, and highlight substantial gene expression divergence both in the long‐term following 150 generations of altered sexual selection intensity and short‐term plastic responses when mating. We found sex‐ and tissue‐ specific effects of sexual selection on gene expression and gene function, alterations in gene expression and gene function specific to origin of the female and male partners, and predicted asymmetric altered gene regulation and function arising from divergent coevolutionary trajectories between sexual selection treatments. Changes in gene expression identified here and in sex‐biased gene expression in response to sexual selection (Veltsos et al., 2017) have recently been shown to be associated with genomic divergence in these lines (Wiberg et al., 2021). Overall, our results complement studies in natural populations in which sexual selection has been implicated in gene expression and genomic divergence.

## AUTHOR CONTRIBUTIONS

The idea was developed by Michael G. Ritchie and Rhonda R. Snook, with funds obtained by Michael G. Ritchie, Rhonda R. Snook and Andrew R. Cossins. The study design was contributed to by Paris Veltsos, Michael G. Ritchie, Rhonda R. Snook, Andrew R. Cossins and Yongxiang Fang. Biological material was prepared by Rhonda R. Snook, Paris Veltsos and Damiano Porcelli. Data were analysed by Yongxiang Fang and Paris Veltsos. The manuscript was written by Paris Veltsos, Michael G. Ritchie and Rhonda R. Snook. The final manuscript was read, edited and approved by all authors.

## CONFLICT OF INTEREST

The authors declare that they have no conflict of interest.

## Supporting information

Supplementary Material

Supplementary Material

## Data Availability

The RNAseq data underlying this article are available in the ArrayExpress repository under accession number  E‐MTAB‐10047 (https://www.ebi.ac.uk/arrayexpress/experiments/E‐MTAB‐10047/). The scripts and output files of the analysis are available at  https://osf.io/z7fm9/.
