# Supplementary material for: Experimental sexual selection reveals rapid evolutionary divergence in sex‐specific transcriptomes and their interactions following mating
Source: Mol Ecol. 2022 Apr 28;31(12):3374–88. doi: 10.1111/mec.16473 (PMC9325514; doi:10.1111/mec.16473)

**Supplemental Information for:**

**Experimental sexual selection reveals rapid evolutionary divergence in sex-specific transcriptomes and their interactions following mating**

Paris Veltsos, Damiano Porcelli, Yongxiang Fang, Andrew R. Cossins, Michael G. Ritchie, Rhonda R. Snook

**Table of Contents:**

| **File S1** | Page 1 |
| --- | --- |
| **File S2** | Page 2 |
| **File S3** | Separate File |

**File S1**: Illustration of the experimental design showing both the long-term selection treatment, and the short-term mating treatment. M: monandry, E: elevated


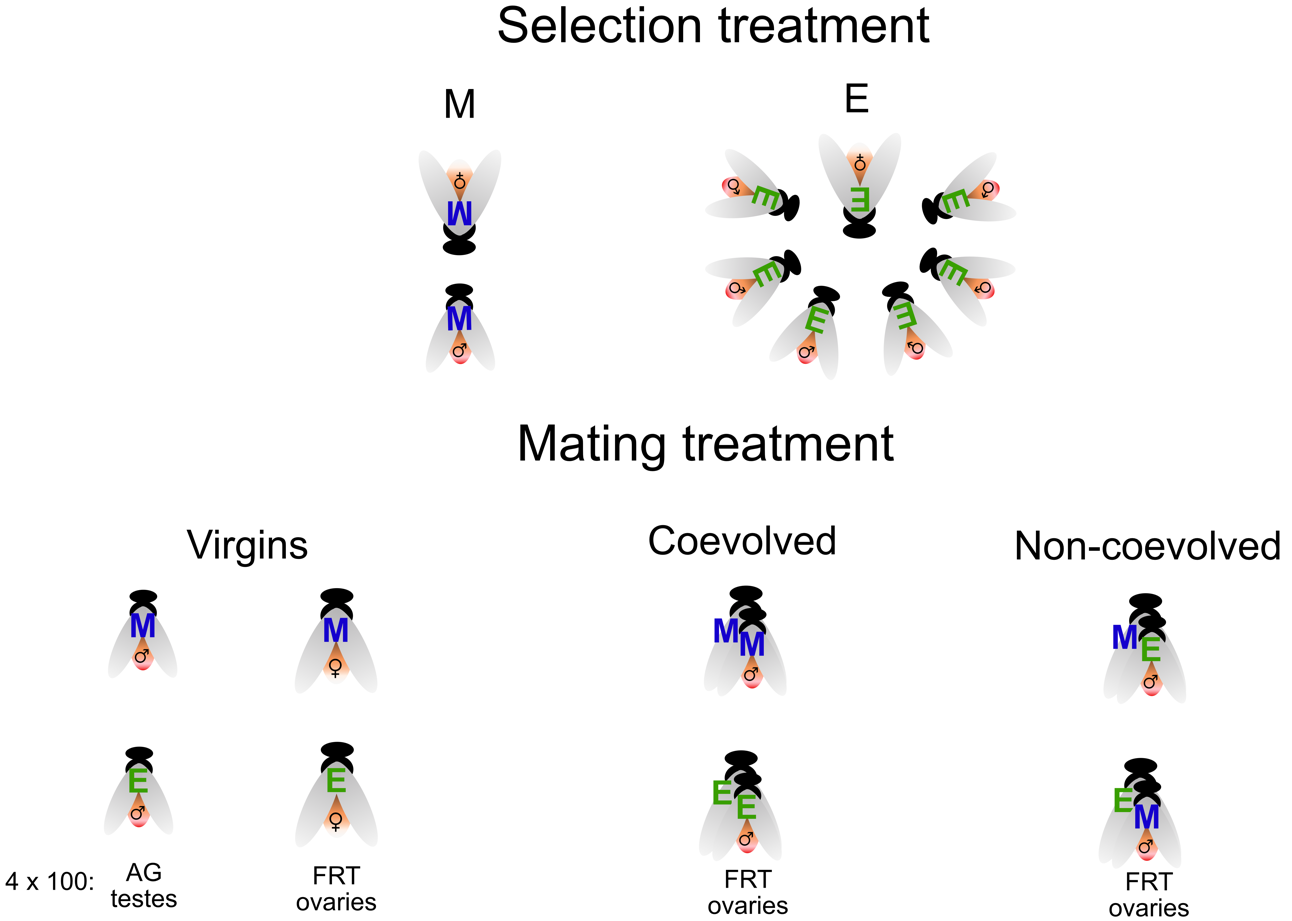


**File S2**: MA plot indicating DE genes in high and low (E vs M) sexual selection contrasts in all tissues for virgin flies. Genes upregulated in E have positive values on the y axis.


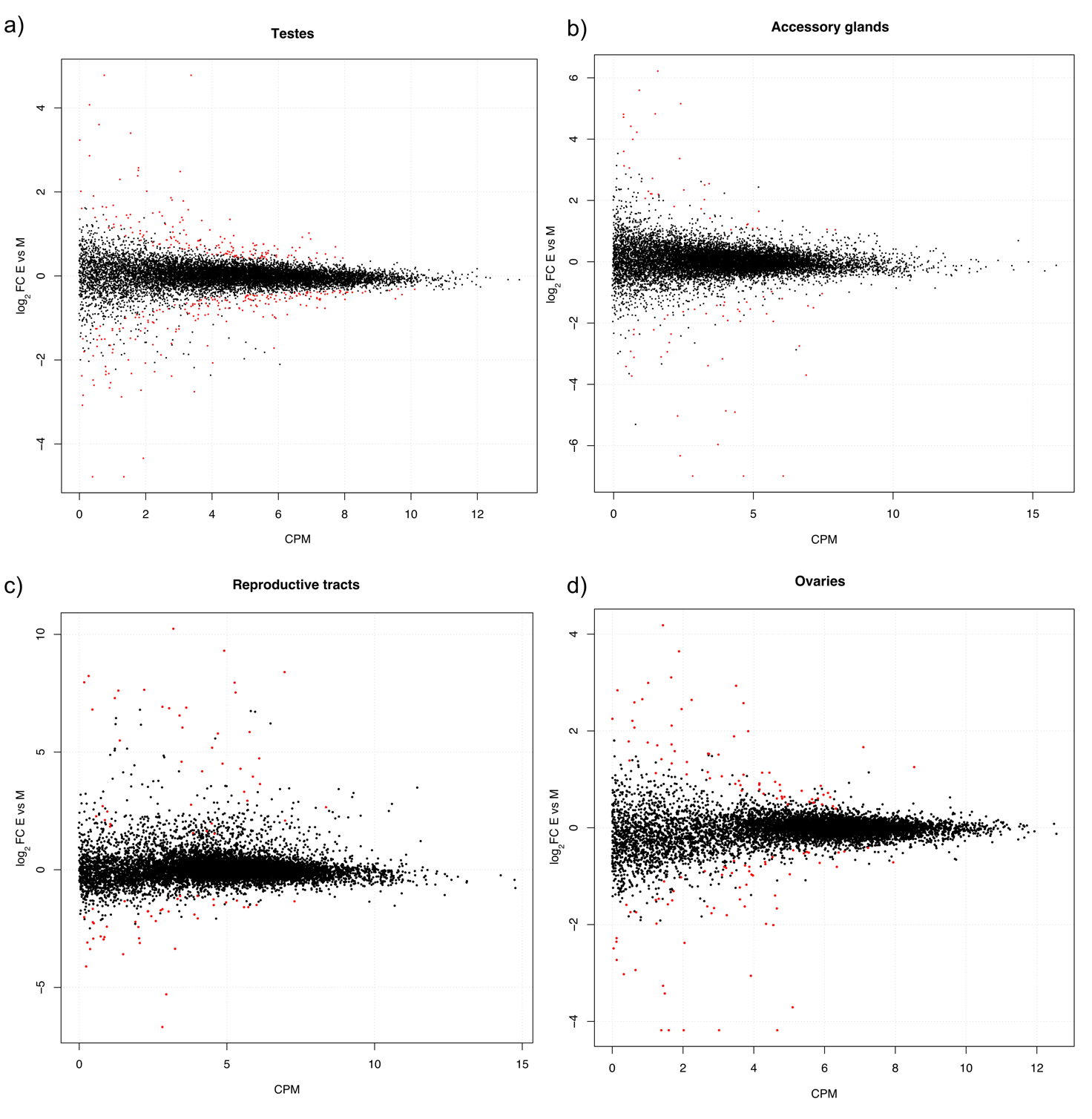

Supplement: Supplementary file 1 — Supplementary Material [file MEC-31-3374-s001.docx]
